# Moderating role of mental health literacy on the relationship between bullying victimization during the life course and symptoms of anxiety and depression in Chinese college students

**DOI:** 10.1186/s12889-023-16326-y

**Published:** 2023-07-31

**Authors:** Xuexue Huang, Yi Zhou, Rong Yang, Danlin Li, Jie Hu, Yanni Xue, Yuhui Wan, Jun Fang, Shichen Zhang

**Affiliations:** 1 MOE Key Laboratory of Population Health Across Life Cycle, Department of Maternal, Child and Adolescent Health, School of Public Health, Anhui Medical University, 81Th Meishan Road, Hefei, 230601 Anhui Province People’s Republic of China; 2 Department of Psychology, Anhui Medical College, 632Th Furong Road, Hefei, 230601 Anhui Province People’s Republic of China; 3 Faculty of Pharmaceutical Science, Sojo University, Ikeda 4-22-1, Kumamoto, 860-0082 Japan; 4 School of Public Health and Health Management, Anhui Medical College, 632Th Furong Road, Hefei, Anhui 230601 People’s Republic of China

**Keywords:** Bullying, Mental health literacy, Anxiety, Depressive symptoms, Students

## Abstract

**Background:**

Exposure to persistent bullying victimization across multiple periods results in a high risk of worse consequences. Although amples studies support the association between bullying victimization and symptoms of anxiety and depression, whether mental health literacy can serve as a moderator on this relationship remains unknown. Therefore, the aim of this study was to examine the patterns of bullying victimization across the life course, and disentangle the moderating effect of mental health literacy between bullying victimization patterns and symptoms of anxiety and depression in Chinese college students.

**Methods:**

A total of 4036 college students were enrolled by cluster sampling from November 2020 to January 2021. Bullying victimization, mental health literacy, and symptoms of anxiety and depression were measured by self-report validated questionnaires. A latent class analysis was applied to identify bullying patterns. The PROCESS program was conducted to analyze whether mental health literacy moderates the link between bullying victimization patterns and symptoms of anxiety and depression.

**Results:**

Three latent patterns of bullying victimization were identified as follows: persistent bullying pattern (6.2%), moderate bullying pattern (10.5%), and low bullying pattern (83.3%). Logisitic regression analysis of anxiety and depressive symptoms indicated that compared with low bullying pattern, persistent bullying pattern had the highest risk. Specifically, mental health literacy moderated the association between bullying victimization pattern and anxiety symptoms (*B* = -0.039, *P* < 0.05).

**Conclusions:**

It is important for practitioners to examine bullying victimization across the life course concurrently rather than a single period in isolation. Interventions and research should enhance mental health literacy to improve the mental health in college students with a history of bullying victimization.

**Supplementary Information:**

The online version contains supplementary material available at 10.1186/s12889-023-16326-y.

## Background

Understanding and addressing mental health during young adulthood is a vital public health issue, as research findings show that the general population of students, typically within the 18–25 age range, present high levels of mental health difficulties [1]. Globally, approximately 31% of college students screened positive for a mental health disorder in 2017 [2]. Specifically, depression and anxiety are significant indicators for mental health and the presence of these symptoms could have a negative effect on individuals if they are not identified early or addressed timely [3]. In the last decade, rising rates of anxiety and depressive symptoms have been observed among college students [4]. The Healthy Minds Network, annually conducting surveys at over 100 colleges and universities, found that the rates of depressive symptoms and anxiety symptoms among college students have raised from 22 and 17% in 2007, to 41% and 34% in 2021 [5]. In addition, a large-scale web-based survey found 25.9% of Chinese college students met the past 2-week diagnostic criteria for patient health questionnaire (PHQ) and 17.8% met the past 12-week diagnostic criteria for a generalized anxiety disorder (GAD) [6]. Depressive symptoms and anxiety symptoms among students may adversely influence their academic performance and quality of life and may contribute to alcohol and substance abuse, decreased empathy, and academic dishonesty [7, 8]. Therefore, it is important to identify modifiable factors that could prevent or alleviate depressive and anxiety symptoms in Chinese college students.

Although the causes of mental health problems are multifactorial, bullying victimization is one risk factor that has received much empirical consideration over the past years [9]. Besides its rampant happen, bullying victimization is of great concern because of the negative health consequences it brings, including physical and psychological symptoms such as sleep disorder, self-harm, lack of appetite, anxiety symptoms, and depressive [10, 11]. Studies have shown that high school students who experienced bullying in the past were more likely to report high depressive symptoms [12, 13]. Furthermore, research has shown that bullying victimization at school can have long-term health effects in adulthood, including increased risk of depression, suicidal ideation, and anxiety disorders [14]. Further, in a recent study conducted in Germany, the results found that adolescents who experienced bullying victimization were more likely to have higher symptoms of depression and anxiety [15]. Collectively, these findings suggest that there may be specific developmental periods when individuals are more likely to develop symptoms of anxiety and depression after exposure to bullying victimization.

Furthermore, exposure to sustained bullying victimization across multiple periods results in a high risk of worse consequences such as externalizing behavior, emotional dysregulation and suicidal behaviors [16]. Currently, many existing studies focused on bullying victimization only during a specific period, mainly in childhood [17], yet the risk of bullying victimization is also high during adolescence and college. The Global School-based Student Health Survey (GSHSS) data suggested that 32.4% of 13–17 years adolescents experienced bullying in 280,076 students [18]. Previous investigations have found that, on average about 20%-25% of students reported non-cyberbullying victimization and 10%-15% reported cyber bullying victimization during college [19, 20]. In China, a study reported that the rates of verbal bullying, relational bullying, physical bullying and cyber bullying among college students in Hefei city were 41.7%, 33.0%, 24.1% and 8.5%, respectively [21]. Addition, a large-scale survey for Chinese colleges students showed that about 8.03% of the participants experienced peer victimization [22]. Meanwhile, a meta-analysis study that included more than 80 projects from different countries, showed that about 35% of students report bullying victimization and 35% report perpetration [23]. A recent study has shown that the consequences of childhood bullying victimization can persist up to midlife and, in addition to mental health, can also impact physical and socioeconomic outcomes, highlighting the need to study the bullying victimization from a life course perspective [24]. In addition, from a life course perspective, individuals may suffer from bullying in all life stages, and victims of bullying might show some specific patterns related to increased risk for depression and anxiety in college students [10, 11]. Understanding the prevalence of bullying victimization during the life course and its health effects is important for preventing bullying. However, few studies have explored this topic.

Both childhood and adolescence are periods of developmental vulnerability during which experiencing bullying victimization has a biological effect on the individual's long-term health trajectory [25]. The Stress Theory Model proposes that psychological stress is a multi-factor process from stressor (bullying victimization) to stress response (depression and anxiety) [26]. Therefore, it is particularly important to reduce the occurrence of stress response by finding moderating factors. Our previous study has found that health literacy has a moderating role on the association between alexithymia and depressive symptoms [27]. As the extension of the concept of health literacy, mental health literacy may also have a moderating effect. Notably, there is a typical extension of health literacy, namely mental health literacy, which is defined as “knowledge and beliefs about mental disorders which aid their recognition, management or prevention” [28]. Inadequate mental health literacy is related to a variety of mental disorders including depressive and anxiety symptoms in college students [29]. Ample studies showed that individuals with inadequate or low levels of mental health literacy tend to have poor mental health outcomes and an increased risk of developing mental health disorders [30, 31]. Meanwhile, low mental health literacy also results in higher mental health stigma, which could ultimately be a barrier to help-seeking [32]. From the existing evidence in the literature, it is clear that increasing mental health literacy is a key to improve mental health outcomes [33, 34].

Although numerous studies support the relationship between bullying victimization and symptoms of anxiety and depression, whether mental health literacy can serve as a moderator on this relationship remains unknown. However, many current studies have focused on the association between a single period of bullying victimization in life course and mental health problems, but did not investigate the interactions and/or combined effects of mutiple periods. Accordingly, current research proposes the following hypothesis: (1) potential patterns of bullying victimization could be observed, (2) patterns of bullying victimization are associated with anxiety and depressive symptoms, and (3) the link between different patterns of bullying victimization and psychological symptoms is moderated by mental health literacy. High levels of mental health literacy will buffer against the effects of bullying victimization on anxiety and depressives symptoms.

## Methods

### Participants and setting

A cross-sectional study was conducted from November 2020 to January 2021, which was approved by the Ethics Committee of Anhui Medical University (approval number 20170290). The study was performed in accordance with the Declaration of Helsinki. Informed consents were obtained from all participants before completing the survey, and all of them could withdraw from the survey at any time without any reason. Data were processed at a restricted location using a personal unidentifiable code for each subject.

Participants were recruited from two medical colleges by a cluster sampling method in Anhui Province, China. Excluding participants who have a history of psychiatric disorders or are being treated with psychiatric medication. A link was given to the students, allowing them to access the electronic questionnaire, including socio-demographic variables, bullying victimization, mental health literacy, depressive symptoms, anxiety symptoms, current cigarette smoking, and alcohol drinking. The students were asked to complete an anonymous questionnaire during 20–30 min session in the classroom. A research staff was responsible for the quality control of the questionnaire to answer the questions from the recipients and to proofread the questionnaire. A total of 4269 participants from sophomores were recruited in this study. Excluding the noncompletion (missing data > 5%) individuals, 4035 valid questionnaires were included, with an effective response rate of 94.5%, which comprised 2019 males and 2016 females with a mean age of 19.26 years (SD = 1.05).

### Measures

The questionnaires consist of questions from demographic variables (i.e., gender, registered residence, any siblings, parents’ educational level, and self-reported family economy), current smoking and drinking status, bullying victimization at specific school-age periods, and the Adolescent Mental Health Literacy Assessment Questionnaire (AMHLAQ) and the Center for Epidemiologic Studies Depression Scale (CES-D), and the Zung Self-Rating Anxiety Scale (SAS), as described below.

We developed the AMHLAQ [35], and used it to evaluate the adolescents’ mental health literacy level of this study. Previous studies suggested good reliability and validity of AMHLAQ for assessing the level of mental health literacy in Chinese university students [36, 37]. The AMHLAQ consists of 22 items grouped into 4 domains, as follows: knowledge (6 items), recognization (5 items), attitude (6 items), and practice (5 items) (see Additional file 1). Each is rated on five selection categories (strongly disagree, disagree, undecided, agree, strongly agree), and the total score is standerdly converted to a score that ranges from 45 to 110, with higher score indicated adequate mental health literacy [35]. Variance cumulative contribution rate was 62.213%; internal consistency test showed that the total questionnaire Cronbach’s α was 0.897, Cronbach’s α of each domain was 0.796 to 0.885; correlation analysis showed that Pearson correlation coefficients of each item and the total score were all above 0.4, and the Pearson correlation coefficient of each item and its domain were more than 0.5; verification results for the measurement model were ^*2*^/*df* = 19.319, RMSEA = 0.069, AGFI = 0.881, NFI = 0.914, RFI = 0.900, CFI = 0.918, and GFI = 0.907, all the indicators were at the significant level and had an acceptable reliability and validity [35]. In this study, internal consistency test showed that the Cronbach’s α coefficient for the AMHLAQ was 0.909, and 0.842, 0.957, 0.939 and 0.934 for four sbuscales, respectively.

SAS and CES-D were used to evaluate the level of anxiety and depressive during the past one week [38, 39]. The SAS survey scales contain 20 questions each. Answers are scored 1–4 points. The standard score was calculated by the total score multiplied by 1.25, which ranges from 25 to 100. Higher scores of CES-D and SAS indicated a higher level of depressive and anxiety symptoms. The CES-D is a commonly used, freely available self-report measure for depressive symptoms, presented in a 4-factor 20-item structure. All CES-D items have 4 response options: rarely or none of the time (< 1 day), some or a little of the time (1–2 days), occasional or moderate of the time (3–4 days), and most or all of the time (5–7 days). Both the SAS and CES-D have been demonstrated to have acceptable validity and reliability in Chinese college students [40, 41]. In this study, the Cronbach’s α coefficient for the CES-D and SAS was 0.861 and 0.770, respectively.

The participants were asked whether they had experienced bulling victimization, including verbal bullying, physical bullying, social bullying and cyber bullying, and the specifics school-age periods, i.e., pre-school, elementary school, junior school, senior high school and college. All students who answered yes were judged as having been bullied.

Cigarette use and alcohol use were elicited in two self-report by answering: “During the past 30 days, on how many days did you smoke cigarettes (including traditional smoking and e-cigarettes)?” and “During the past 30 days, on how many days did you have at least one drink of alcohol?”. where 0 day was coded as “no smoking and alcohol” and other option coded as “smoking and alcohol” [42].

### Statistical analysis

Statistical analysis was carried out by SPSS 23.0 (SPSS Inc, Chicago, IL) and Mplus (Mplus Version 7.4). Statistical analyses were carried out in four main steps. In step 1, a latent class analysis was applied to identify patterns of bullying experiences. In step 2, Mann–Whitney U Test and Kruskal–Wallis H Test were used to compare the distribution of anxiety and depressive symptoms with different characteristics. In step 3 spearman’s correlation was used to test the associations between mental health literacy, patterns of bullying victimization and symptoms of anxiety and depression. In step 4, according to Baron and Kenny (1986) [43], the PROCESS program of moderation was used to perform a moderation analysis. In SPSS PROCESS the interacting effect is calculated automatically via the software and it also produces the proportion of the variance explained by the moderating effect of mental health literacy (*R*^*2*^ increases due to interaction). We adjusted the key sociodemographic correlates (gender, self-reported family economy, parents’ educational level, smoking, drinking and any sibling) in the moderation model. We explored whether mental health literacy moderated the relationship between bullying patterns and symptoms of anxiety and depression. When the 95% *CI* did not contain zero, the moderating effect was considered significant.

Latent class analysis (LCA) is a methodological approach that helps to explain population heterogeneity within observed data through the identification of underlying subgroups of individuals, thus allowing examination of bullying experiences in different times while dealing with diverse nature of population [44]. The best-fit number of classes was determined based on a set of indexes including the.

Akaike Information Criterion (AIC), Bayesian Information Criterion (BIC), adjusted Bayesian Information Criterion (aBIC), Lo-Mendell-Rubin (LMR), Bootstrapped Likelihood Ratio Test (BLRT), and Entropy measures [45]. A lower value of AIC, BIC and aBIC indicate better model fit [46]. For the LMR and BLRT, significant *P* value (*P* < 0.05) signifies that the model fit is better than the model with one less classification [47]. Moreover, the entropy value is closer to 1, the clearer the model separation [48].

## Results

### Latent class analysis of bullying victimization

Table 1 describes fit statistics for one to five class models. The five classes of models did not replicate the optimum log-likelihood value and were therefore not considered further. Based on the lower AIC value, the lowest BIC value and aBIC value, three kinds of solutions are selected as the final best fitting model. Moreover, there was no significant difference in the *P*-value of LMR in the fourth group. For model 3, the bootstrap verifier also proves that it has a good fit (*P* < 0.001). Therefore, we focus on model 3 in the following study, and all remaining results are reported for three types of solutions.

**Table 1:** Model fit statistics for each of the fitted latent class analysis models

Classes	*df*	AIC	BIC	aBIC	LMR-LRT	BLRT	Entropy	Classification probability
2	11	9396.322	9465.653	9430.699	< 0.0001	< 0.0001	0.863	0.09765 0.90235
**3**	**17**	**9352.158**	**9459.305**	**9405.287**	** < 0.0001**	** < 0.0001**	**0.848**	**0.05626** **0.10235** **0.84139**
4	23	9356.262	9501.226	9428.142	0.5616	0.5694	0.771	0.10012 0.05155 0.01512 0.83321
5	29	9360.035	9542.815	9450.666	0.5119	0.5173	0.821	0.04758 0.07410 0.01264 0.02379 0.84188

*df* degrees of freedom, *AIC* Akaike Information Criteriam, *BIC* Bayesian Information Criteria, *aBIC* Adjusted Bayesian Information Criteria, *LMR-LRT* Lo-Mendell-Rubin Likelihood Ratio, *BLRT* Bootstrapped Likelihood Ratio Tests Bolded row represents the selected model

**Fig. 1:**
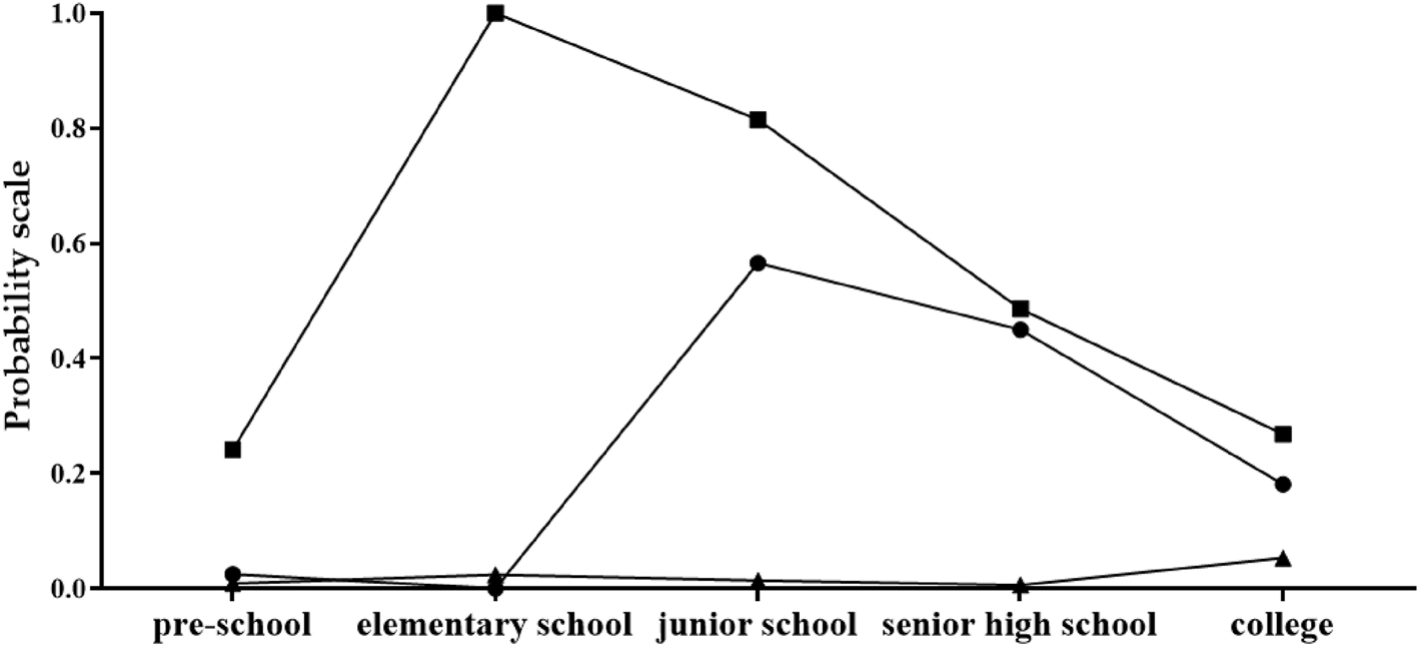
Different stages of bullying experiences of the best-fitting three-class pattern persistent ■ bullying pattern; ● moderate bullying pattern; ▲ low bullying pattern

Figure 1 showed the three identified latent patterns. Latent class 1 was defined as a “low bullying” pattern which includes 83.3% (*n* = 3360) of the samples. In this class, few students reported bullying victimization, with 0.9% of pre-school, 2.4% of elementary school, 1.4% of junior school, 0.6% of senior high school, and 5.3% of college. In contrast, latent class 3 was labelled as a “persistent bullying” pattern with 6.2% (*n* = 251) of the samples, in which a majority of students reported bullying victimization: 24.1% of pre-school, 81.5% of junior school, 48.6% of senior high school, and 26.8% of college, and all of them had bullied at elementary school (100%). In addition, latent class 2 was characterized by reported fewer bullying victimization at pre-school (2.5%) and elementary school (0%), but higher percentages of bullying victimization at junior school (56.6%), senior high school (45.0%), and college (18.1%), which was labelled as a “moderate bullying” pattern that includes 10.5% (*n* = 425) of the samples.

### Description of anxiety and depressive symptoms

Table 2 presented the scores of anxiety and depressive symptoms by general demographic characteristics. Female college students had higher scores of anxiety and depressive symptoms than male college students (*χ*^*2*^ = -4.903 and -8.318, *P* < 0.001 for each), and higher anxiety and depressive symptoms scores were also found in students of low family income, having cigarette and alcohol use (*P* < 0.001 for each). Meanwhile, the results showed significant associations of any sibling (*χ*^*2*^ = -3.007, *P* = 0.003) with anxiety symptoms. In addition, there was a marked difference between bullying patterns with the scores of depressive symptoms [low bullying [9] vs. moderate bullying [12] vs. persistent bullying [13], *Z* = 239.027, *P* < 0.001)] and anxiety symptoms [low bullying [40] vs. moderate bullying [43] vs. persistent bullying [44], *Z* = 111.116, *P* < 0.001)].

**Table 2:** Participant characteristics in the current study

Variable	*n (%)*	Depressive symptoms					Anxiety symptoms				
		*M*	*P*_*25*_	*P*_*75*_	*χ*^*2*^/*Z*	*P-value*	*M*	*P*_*25*_	*P*_*75*_	*χ*^*2*^/*Z*	*P-value*
Gender					-4.903	< 0.001				-8.318	< 0.001
Male	2019(50.1)	9	4	12			39	33	44		
Female	2016(49.9)	12	5	12			44	35	44		
Registered residence					-1.041	0.298				-0.163	0.871
Rural	2494(61.8)	10	4	12			41	34	44		
Urban	1541(38.2)	10	4	12			41	34	44		
Any siblings					-0.175	0.865				-3.007	0.003
Yes	2978(73.8)	10	4	12			40	34	44		
No	1057(26.2)	11	4	12			43	35	44		
Father’s educational level					-0.868	0.386				-0.655	0.512
< High school degrees	2971(73.6)	10	4	12			41	34	44		
≥ High school degrees	1064(26.4)	10	4	12			41	33	44		
Mother’s educational level					-1.013	0.311				-0.546	0.585
< High school degrees	3336(82.7)	10	4	12			41	34	44		
≥ High school degrees	699(17.3)	10	4	12			43	33	44		
Self-reported family economy					22.122	< 0.001				23.317	< 0.001
Bad	1463(36.3)	11	5	13			43	35	44		
Normal	2426(60.1)	10	4	12			40	34	44		
Good	146(3.6)	9	5	12			40	33	44		
Cigarette use					-4.097	< 0.001				-6.510	< 0.001
No	3732(92.5)	10	4	12			40	34	44		
Yes	303(7.5)	12	6	13			44	38	44		
Alcohol use					-5.293	< 0.001				-5.344	< 0.001
No	3527(87.4)	10	4	12			40	34	44		
Yes	508(12.6)	12	6	13			44	35	44		
Pattern of bullying experience					239.027	< 0.001			111.116	< 0.001	
Low bullying	3360(83.3)	9	4	12			34	44			
Moderate bullying	424(10.5)	12	7	18			36	46			
Persistent bullying	251(6.2)	13	7	23			36	54			

Statistical methods: *M* median, *P*_*25*_ lower quartile, *P*_*75*_ upper quartile, Mann–Whitney U Test and Kruskal–Wallis H Test were used to compare the distribution of depressive symptoms and anxiety symptoms with different characteristics

### Correlation among of bullying patterns, mental health literacy and symptoms of anxiety and depression

Correlation analysis between key variables were presented in Table 3. Spearman correlation analysis revealed significant correlations between mental health literacy and depressive symptoms (*r* = -0.390, *P* < 0.001), anxiety symptoms (*r* = -0.519, *P* < 0.001). Meanwhile, anxiety symptoms (*r* = 0.146) and depressive symptoms (*r* = 0.215) were positively correlated with bullying patterns (*P* < 0.001 for each).

**Table 3:** Correlations among variables in the measurement model

Measure	Mental health literacy	Depressive symptoms	Anxiety symptoms	Pattern of bullying experience
Mental health literacy	1.000			
Depressive symptoms	-0.390^***^	1.000		
Anxiety symptoms	-0.519^***^	0.694^***^	1.000	
Pattern of bullying experience	-0.012	0.215^***^	0.146^***^	1.000

Statistical methods: Spearman’s correlation analyses; ^***^P < 0.001

### Moderating effects of mental health literacy in the association between bullying patterns and symptoms of anxiety and depression

**Fig. 2:**
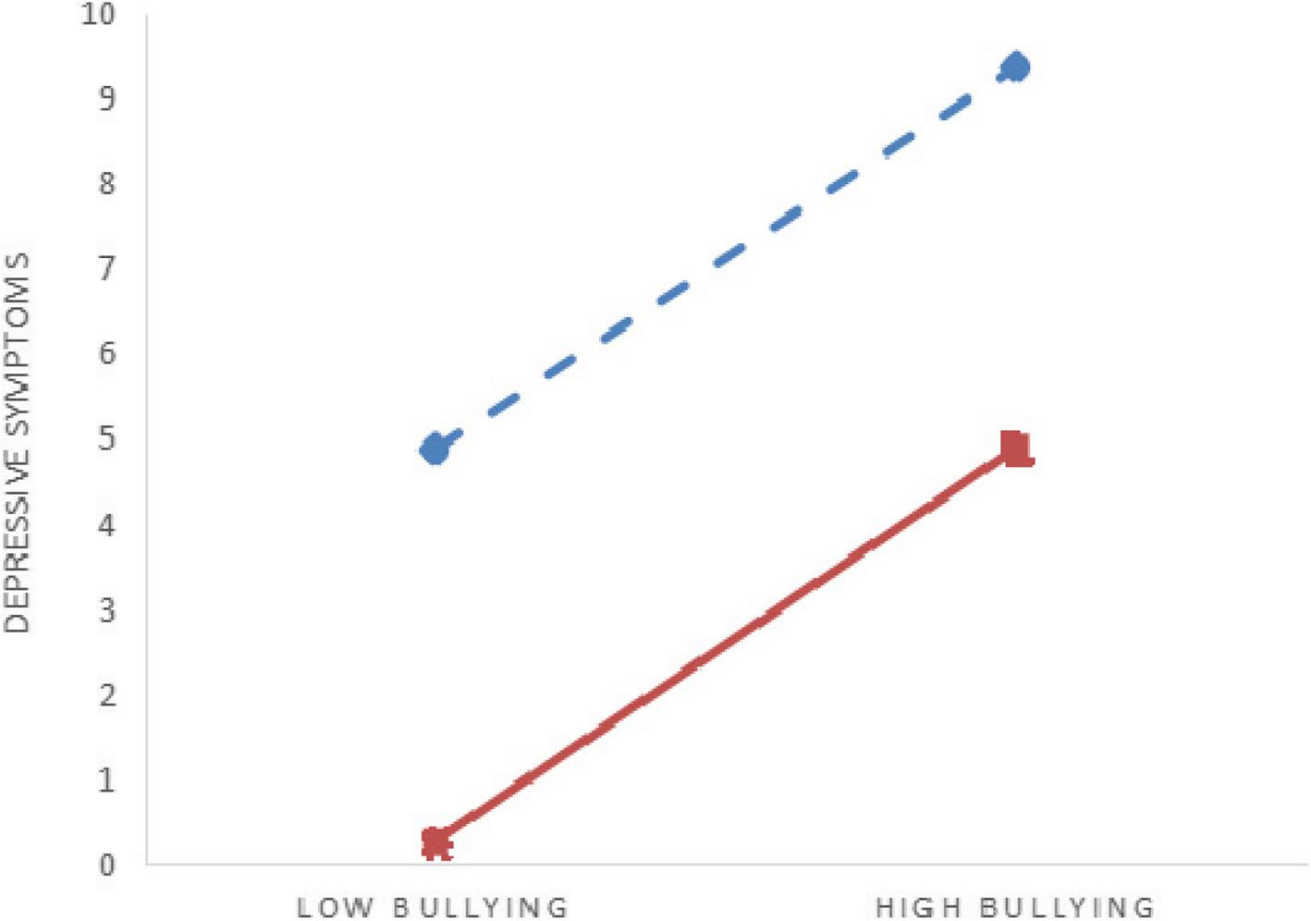
Moderating effects of mental health literacy between life course bullying experience and  depressive symptoms.  high mental health literacy  low mental health literacy

**Fig. 3:**
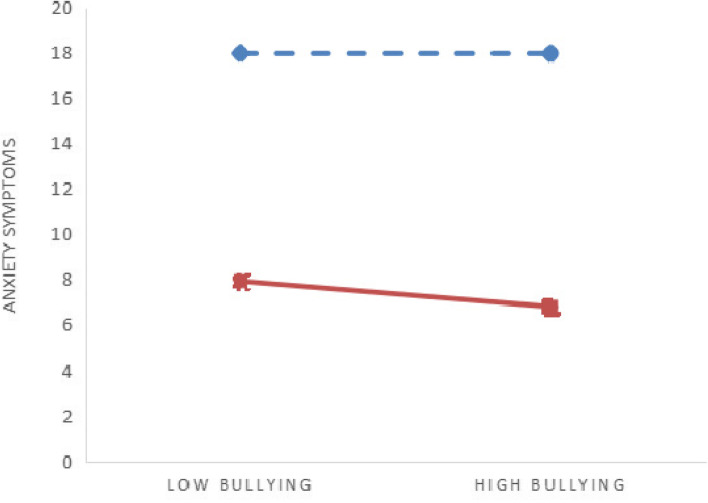
Moderating effects of mental health literacy between life course bullying experience and anxiety symptoms.  high mental health literacy  low mental health literacy

As shown in Table 4, mental health literacy moderated the association between bullying patterns and anxiety symptoms (*B* = -0.039, *P* < 0.05), while mental health literacy did not moderate the association between bullying patterns and depressive symptoms (*B* = 0.005, *P* > 0 0.05). To further verify the moderating effects, moderating effects diagrams were drawn for different mental health literacy levels (low, high). The slope of the straight line reflected the impact effect sizes of bullying on depression and anxiety (see Figs. 2 and 3). Simple slope test showed that for both low and high mental health literacy, college students showed a significant upward trend in depressive symptoms as bullying increased (simple slope = 4.100, *t* = 3.591, *P* < 0.001; simple slope = 4.230, *t* = 2.758, *P* < 0.01). At the same time, with the increase of bullying, the change trend of anxiety symptoms of college students was not significant, and bullying could not significantly predict the anxiety symptoms of college students (simple slope = -0.016, *t* = -0.013, *P* = 0.990; simple slope = -1.028, *t* = -0.642, *P* = 0.521). Thus, mental health literacy plays a moderating role in the relationship between bullying and anxiety (Fig. 3).

**Table 4:** Moderating effects of mental health literacy between life course bullying experience and symptoms of anxiety and depression

Variable	Depressive symptoms					Anxiety symptoms				
	*B*	*SE*	*t*	*LLCI*	*ULCI*	*B*	*SE*	*t*	*LLCI*	*ULCI*
Pattern of bullying experience	3.736	0.211	17.750^***^	3.324	4.149	2.825	0.221	12.809^***^	2.392	3.257
Mental health literacy	-0.189	0.009	-21.153^***^	-0.206	-0.171	-0.300	0.009	-32.130^***^	-0.318	-0.282
Moderating effects	0.005	0.016	0.302	-0.027	0.037	-0.039	0.017	-2.281^*^	-0.072	-0.006
Registered residence	-0.001	0.165	-0.005	-0.324	0.322	0.034	0.173	0.195	-0.305	0.372
Any sibling	0.006	0.293	0.022	-0.568	0.580	-0.361	0.307	-1.176	-0.962	0.241
Father’s educational level	-0.151	0.305	-0.496	-0.750	0.447	-0.118	0.320	-0.370	-0.745	0.509
Mother’s educational level	0.385	0.353	1.091	-0.307	1.076	0.400	0.370	1.082	-0.325	1.124
Self-reported family economy	-0.842	0.221	-3.817^***^	-1.274	-0.410	-0.712	0.231	-3.083^**^	-1.165	-0.259
Cigarette use	0.202	0.609	0.333	-0.991	1.400	1.161	0.637	1.821	-0.089	2.411
Alcohol use	1.863	0.421	4.423^***^	1.037	2.689	1.523	0.441	3.451^***^	0.658	2.389
Gender	0.727	0.275	2.647^**^	0.189	1.266	0.617	0.288	2.143^*^	0.053	1.181
*R*^*2*^, *F*	*R*^*2*^ = 0.174, *F* = 77.044^***^					*R*^*2*^ = 0.246, *F* = 119.410^***^				
*ΔR*^*2*^, *ΔF*	*ΔR*^*2*^ < 0.001, *ΔF* = 0.092					*ΔR*^*2*^ = 0.001, *ΔF* = 5.196^*^				

*LLCI* lower level cofidence interval, *ULCI* upper level cofidence interval, *ΔR*^*2*^ R^2^change due to interaction, *ΔF* Fchange due to interaction

^*^P < 0.05

^**^P < 0.01

^***^P < 0.001

## Discussion

In this study, we explore the latent patterns of college students’ bullying victimization experiences in a large Chinese sample. Meanwhile, as far as we know, this is the first study examined the moderating effects of mental health literacy in the association between bullying patterns and symptoms of anxiety and depression. This patterning helps to link a life course of bullying victimization experiences. Consistent with previous research examining bullying patterns among college students [49], the present study found three categories, although the types and indicators of bullying victimization experiences are not completely consistent and the age range of the respondents are different. It is worth noting that the moderate bullying pattern (10.5%) who reported higher prevalence of bullying victimization in junior school, senior high school and college, and these results showed that bullying victimization predominantly occurs in pre-college, and reached a relative peak within middle school, consistent with previous study [49]. Besides, the least number of students belonged to persistent bullying pattern (6.2%), having higher rates of bullying victimization in all periods. However, given China’s population base, it is necessary to identify and intervene persistent pattern.

In addition, we found a cumulative effect between the pattern of bullying victimization experiences and symptoms of anxiety and depression, which was consistent with previous findings that exposure to sustained bullying victimization in multiple periods may result in a high risk of worse consequences [49]. This finding supports an Accumulation of Risk Model, which posits that chronic exposure is related to an increased risk of poor health outcomes in a dose–response manner, irrespective of timing [50]. Furthermore, we found that female students had higher symptoms of anxiety and depression, which may because women are considered to be more perceptual, emotional, and relatively vulnerable to tension [51]. Our results indicated anxiety symptoms and depressive symptoms were associated with bad family economic status, cigarette and alcohol use, which was similarly reported in previous studies [52, 53].

Our results indicated that patterns of bullying victimization experience had negative effects on college students’ mental health, that is, bullying patterns is positively associated with anxiety and depressive symptoms, this finding is consistent with previous studies [54, 55]. Bottino et al. suggested the adverse effects of early bullying victimization on college students’ mental health, with a clear temporal ordering of exposure and outcomes, the feelings of helplessness and powerlessness to defend themselves from incidents of bullying victimization can increase the sense of fear and emotional distress, contributing to the emergence of mental health problems [56]. Moreover, bullying victimization and symptoms of anxiety and depression may be bidirectionally linked. These mental distress symptoms may, in turn, contribute to life stress among adolescents, including (re) bullying. Adolescents with mental health problems may lack adaptive coping, positive interpersonal skills, and supportive peer relationships, which may increase their problem internet use and risk of experiencing bullying victimization [57]. Future research using prospective cohort studies is needed to elucidate the bidirectional relationships between bullying victimization and these mental health problems.

Furthermore, the association between mental health literacy and psychological symptoms is equally acknowledged. College students having inadequate mental health literacy are at risk of exhibiting anxiety and depressive symptoms [37]. Namely, if the mental health literacy of college students could be improved, positive developmental outcomes will thus be achieved with lower levels of psychological symptoms in college students. Meanwhile, previous researches suggested that college students, especially those with a history of bullying victimization and inadequate mental health literacy, seem to be a vulnerable group for anxiety and depressive symptoms, which is consistent with previous studies [36, 54]. These findings further support the importance of the adequate mental health literacy for protecting symptoms of anxiety and depression. Namely, individuals with adequate mental health literacy are more likely to acquire risk of bullying victimization, which may in turn decrease their level of anxiety [58]. In addition, college students with adequate mental health literacy might have been more likely to seek help from their parents or teachers, and to communicate with their parents or teachers when negative experiences (such as bullying victimization) occurred [32]. Consequently, parents or teachers who are aware of such victimization will thus provide timely interventions. Also, these students have been more likely to seek and obtain emotional support and/or problem-solving from parents or teachers directly after and/or during their experience of bullying victimization.

The main contribution of this study is to demonstrate what extent different bullying patterns are associated with symptoms of anxiety and depression through mental health literacy. To our knowledge, our study is the first to examine whether the mental health literacy moderate the associations between patterns of bullying victimization experience and symptoms of anxiety and depression in Chinese medical students, which may help to understand the potential negative effects of low mental health literacy and different bullying patterns, and to design targeted intervention programs to promote college students’ mental health. However, there are some limitations in this study. Firstly, due to the cross-sectional nature of this study, no direct evidence of causal association can be obtained, so further longitudinal research is needed to confirm. Secondly, we used self-reported data, which may lead to certain bias in the information. Finally, the subjects were from two medical schools, so it is prudent to generalize our findings to all Chinese college students.

## Conclusion

The present study illustrates the possible association of anxiety and depression, patterns of bullying victimization experiences, and mental health literacy in the context of Chinese culture. Exposure to sustained bullying victimization across multiple periods results in a high risk of worse health outcomes, so when an interventions for the prevention of anxiety and depressive symptoms is designed among college students, patterns of bullying victimization experiences should be taken into account. In addition, most previous studies on bullying victimization and anxiety simply focused on the association between them but did not consider the role of mental health literacy on them, even though the importance of mental health literacy has attracted more and more attentions. Based on the findings in the present study, it is clear that enhancing individual’s level of mental health literacy is important to reduce their anxiety. Despite the priority efforts focused on decreasing bullying victimization in previous interventional measures, we consider improving mental health literacy may also be a focus. The findings strongly indicate the need of enhancing mental health literacy to improve the mental health in college students with a history of bullying victimization.

## Supplementary Information

**Additional file 1.**

## Data Availability

The datasets generated and/or analysed during the current study are not publicly available, but are available from the corresponding author on reasonable request.
