# Supplementary material for: Moderating role of mental health literacy on the relationship between bullying victimization during the life course and symptoms of anxiety and depression in Chinese college students
Source: BMC Public Health. 2023 Jul 31;23:1459. doi: 10.1186/s12889-023-16326-y (PMC10388468; doi:10.1186/s12889-023-16326-y)
Supplement: Supplementary file 1 — Additional file 1. [file 12889_2023_16326_MOESM1_ESM.pdf]

Adolescent Mental Health Literacy Assessment Questionnaire (AMHLAQ)

(Please select only one answer.)

Items	Strongly disagree	Disagree	Undecided	Agree	Strongly agree
1. Suffering from psychosomatic trauma (e.g.emotional, physical abuse, etc.) may lead to mental illness.					
2. Traumatic brain injury or brain disease may cause mental illness.					
3. The duration of symptoms of psychophysiological disorders is an important factor in the diagnosis of mental illness.					
4. Psychological consultation is an effective way to treat mental illness.					
5. Increase of communication with others contributes to mental health.					
6. A balanced diet contributes to mental health.					
7. Loss of interest and happiness is a major symptom of depression.					
8. Anxiety patients often have unexplained worry, excessive nervousness and fear.					
9. People with obsessive-compulsive disorder usually have repeated obsessive thoughts or behaviors (e.g. washing hand repeatedly).					
10. People with social phobia usually become extremely nervous and anxious when communicating with others or participating in group activities.					
11. People with post-traumatic stress disorder often try to avoid the situations and content related to trauma.					
12. If I had a mental disorder, I would feel ashamed.					
13. If my relatives or friends had mental illness, I would feel ashamed.					
14. If my relatives or friends had mental illness, I wouldn't to help her/him.					
15. I think people with mental disorders(illness) usually come from					

low-income families.

16. I don't think mental disorders (illness) can affect people's feelings.

17. I think only adults can suffer from mental illness.

18. I can control my negative emotions and bad behaviors.

19. I can solve my current psychological distress.

20. If my relatives or friends had mental illness, I would encourage her/him to seek help.

21. If my relatives or friends had mental illness, I would give support to her/him.

22. If my relatives or friends had mental illness, I would listen to her/him without judging or criticizing.
